# High Temperature and Ethinylestradiol May Reduce Body Growth, Liver and Hepatocyte Volumes and Lipid Droplets in Adult Male Guppies

**DOI:** 10.3390/ani15142152

**Published:** 2025-07-21

**Authors:** Margarida Vilaça, Sukanlaya Tantiwisawaruji, Maria João Rocha, Eduardo Rocha

**Affiliations:** 1 Laboratory of Histology and Embryology, Department of Microscopy, School of Medicine and Biomedical Sciences (ICBAS), University of Porto (U. Porto), 4050-313 Porto, Portugal; up201804847@edu.icbas.up.pt (M.V.); mjrocha@icbas.up.pt (M.J.R.); 2 Group of Animal Morphology and Toxicology, Interdisciplinary Centre for Marine and Environmental Research (CIIMAR/CIMAR), University of Porto (U. Porto), 4450-208 Porto, Portugal; sukanlaya.tan@kmutt.ac.th; 3 Learning Institute, King Mongkut’s University of Technology Thonburi, Bangkok 10140, Thailand

**Keywords:** estrogens, fish, hepatocytes, liver, warming, stereology

## Abstract

Global warming raises surface water temperatures, impacting fish alongside pollutants, such as ubiquitous xenoestrogens. Combined stressor effects are poorly studied but likely to worsen impacts and hinder biota adaptation, warranting further research. Unadapted fish face heightened risks. The liver is a vital metabolic organ, sensitive to temperature and xenoestrogens, eventually adjusting hepatocyte size and number to ensure survival, growth, and reproduction. This study assessed, for the first time, the impact of exposure (45 days) to thermal stress (29 °C versus 26 °C) and ethinylestradiol (EE2, 5 ng/L) on male guppies, primarily on body and quantitative liver morphology. Higher temperature reduced body mass (14%) and standard length (3.6%) gain. EE2 exposure reduced body mass increase (14%), hepatosomatic index (20%), and the volumes of the liver (32%), hepatocytes (16%), and their nuclei (17%). The nucleus-to-cytoplasm ratio and total hepatocyte number remained stable. No histopathological lesions existed. Guppies appear to have adapted to stressors by reducing hepatocyte size and utilizing lipid reserves, yet they exhibited deficits in body growth and hepatosomatic index. Gonadal maturation was unaffected. Only under EE2 at 29 °C did hepatocytes show minimal lipid droplet content (less vacuolation). This indicated exhausted reserves, reinforcing how heat and toxicants interact to exacerbate impacts.

## Simple Summary

Human actions are making rivers and lakes warmer and adding more pollution by chemicals, including a synthetic hormone called ethinylestradiol (EE2), which comes from birth control pills. This means that fish often face both warmer water and chemical pollution at the same time. In this study, for 45 days, scientists investigated how these two stress factors affect male guppies—small tropical fish often kept in home aquariums. The researchers found that warmer water (3 °C higher than usual) caused the guppies to weigh less and grow shorter. EE2 in the water also caused the guppies to gain less body weight and affected their liver size (with smaller liver cells than in unexposed fish) and internal microscopic structure, though their livers appeared healthy without visible damage. Even under stress, the guppies seemed to cope by using up fat stored in their liver cells. They were even able to continue to produce sperm cells. However, the combined effects of heat and EE2 exacerbated the fat loss. This suggests that while guppies exhibit signs of adaptation, these environmental pressures continue to challenge their health and growth. It is thus justified to further study the long-term impacts.

## 1. Introduction

Climate change is one of the planet’s most pressing challenges, owing to the massive amounts of greenhouse gases released into the atmosphere since the Industrial Revolution [1]. Global warming, which raises water temperatures, harms aquatic ecosystems and fisheries services by disrupting the temperature-dependent functioning of these habitats [2]. Most organisms in these habitats, including fish, are ectothermic; therefore, global warming directly affects their metabolism by increasing their body temperature [3]. In fish, during the initial phase, the energy for metabolic processes increases, improving them and eventually stimulating growth [4]. Logically, this only happens if the temperature rise falls within a species’ tolerance and survival spectrum. However, even under these conditions, a long-term elevation of metabolic rate cannot be sustained by a corresponding increase in cardiac and respiratory rates [5,6]. Additionally, higher temperatures may cause protein denaturation and functional loss, ultimately impairing animal growth [5,7] and potentially inducing population impacts [7,8]. These effects can begin in early life stages, as clearly illustrated by the long-term impacts of temperature—modulated by pollution levels—on ichthyoplankton composition and abundance [9]. Ultimately, global warming threatens fish, from individuals to populations [2,10,11].

The fish liver is the primary metabolic organ regulating the body’s energy metabolism. Its structure and function can be influenced by temperature. For example, Liu et al. (2016) [12] described how, besides increasing the hepatic lipidic and glycogenic contents in the Wuchang bream (*Megalobrama amblycephala*), hyperthermia (32 °C) altered the structure of the hepatocyte nucleus and mitochondria. The authors also noticed increased oxidative stress, accompanied by higher expression of heat shock proteins. In the same vein, Nuez-Ortín et al. (2018) [13] confirmed, by proteomics, the influence of warmer temperatures on the hepatic metabolism of Atlantic salmon (*Salmo salar*): protein synthesis was suppressed at 21 °C, energy production became more dependent on amino acids (instead of glucose or fatty acids), reverse transport of cholesterol and its catabolism intensified, oxidative stress increased, and the hepatocytes’ cytoskeleton was destabilized.

Another global problem is water pollution, the impacts of which can be studied using various methodological tools and biological matrices, such as biochemistry [13], histology [12], and hematology [14]. These provide biomarkers of exposure and pollutant effects, while also monitoring other environmental changes and assessing fish health. Among the many aquatic pollutants, endocrine disruptor chemicals (EDCs) are ubiquitous micropollutants of emerging concern [15,16]. EDCs are natural or synthetic compounds of anthropogenic origin that interfere with the normal functioning of the endocrine system [17]. Synthetic EDCs encompass a wide range of substances, including solvents, lubricants and their derivatives, plastic components, pesticides, fungicides, and drugs [15,17]. Awareness about such EDCs has increased because they are hard to degrade and can persist for decades in aquatic ecosystems [16,18]. Additionally, EDCs are lipophilic and, therefore, prone to bioaccumulation and biomagnification [18]. For animals higher in the food chain, the problem of bioaccumulation is more severe [19]. Moreover, when organisms degrade some EDCs, more toxic compounds may form [17], amplifying the exposure outcomes.

EDCs deserving of attention for decades are xenoestrogens, including natural substances like phytoestrogens and mycoestrogens, medicines such as 17α-ethinylestradiol (EE2) and progestins in oral contraceptives, and compounds from industrial products like bisphenol A and tributyltin [20]. In the present study, EE2 is the EDC of interest due to its potent estrogenic activity and widespread presence, which is estimated to contaminate at least 65% of surface waters worldwide [21]. Moreover, average concentrations in surface waters of the “top ten countries” range from a few ng/L to 28 ng/L, with the EE2-equivalent 17β-estradiol (E2) activity ranging from 0 to 33 ng/L [21]. When other xenoestrogens are added to EE2, the total estrogenic potency can easily exceed 50 ng/L and reach up to 198 ng/L of EE2 equivalents (EE2eq) [15]. The detrimental effects of xenoestrogens on fish liver have been reported, primarily associated with “feminization” in exposed males or juveniles, resulting in the organ producing and exporting abnormally high levels of zona radiata proteins and the yolk precursor lipoprotein vitellogenin (VTG) [22]. The liver impacts can be functional and structural. Kinnberg et al. (2003) [23] reported increased vacuolation of hepatocytes in offspring of female guppy (*Poecilia reticulata*) exposed to octylphenol and E2. Cakmak et al. (2006) [24] unveiled that E2 diminished glycogen and protein contents and increased lipid (namely triglycerides) levels in male rainbow trout (*Oncorhynchus mykiss*) liver. In EE2-injected male platyfish (*Xiphophorus maculatus*), immunohistochemistry confirmed the induction of VTG expression, with hepatocytes appearing more basophilic and vacuolated [25]. In addition to histological changes, fish hepatocytes can proliferate in response to estrogens [26,27], although not consistently [25]: a poorly understood phenomenon that may contribute to adaptive liver hypertrophy.

The independent effects of temperature and exposure to EDCs have been characterized, but a significant caveat remains in understanding their combined effects, especially on the fish liver under EE2 exposure. DeCourten et al. (2017) [28] explored the co-effects of exposure to EE2 or the pesticide bifenthrin at a high temperature (28 °C) along three generations (F0, F1, and F2) of the estuarine fish inland silverside (*Menidia beryllina*). They concluded that the highest temperature, combined with EE2, disrupted sex determination and adaptive development in F1. Moreover, F2 exhibited fewer viable descendants and a higher incidence of congenital malformations. Cardoso et al. (2017) [29] showed that the progestin levonorgestrel (LNG) diminished the ovary maturation degree of the zebrafish (*Danio rerio*), which was more pronounced at the highest temperature (30 °C). The same study evaluated the impact of increased temperature on the effects of LNG on the liver of female zebrafish, noting that hepatocytes from animals exposed to LNG at 30 °C had more glycogen. Moreover, hepatocytes showed reduced VTG load when the lowest LNG concentration was combined with 30 °C. Jacquin et al. (2019) [30] pointed in the same direction with the goldfish (*Carassius auratus*), as the highest temperature (32 °C) exacerbated toxicity; the synergistic effect of temperature and pesticide exposure led to apoptosis, inflammation, and necrosis of the liver and gill. Cox et al. (2018) [31] evaluated in the freshwater fish fathead minnow (*Pimephales promelas*) the influence of temperature on the effects of estrone (E1). It was concluded that at 15 °C, the production of VTG was higher than at 18 and 21 °C and that the hepatocytes were more vacuolated at 15 and 18 °C. Control males presented lower VTG plasmatic levels than those exposed to E1 from 15 to 21 °C. The differences between control and exposed males were more distinct at 15 °C.

Founded on the considerations above and using the guppy as a model organism, we hypothesize that subchronic exposure to environmental levels of EE2 and a sub-optimal elevated temperature, independently or in combination, may affect male body biometry (viz., mass, length) and liver mass and histology through changes in hepatocyte structure, size and/or cellularity. To test the hypothesis, adult male guppies were exposed to 5 ng/L of EE2 for 45 days at 26 and 29 °C. Subsequently, the liver was studied by light microscopy and design-based stereology. For correlative purposes, the gonadal status was confirmed. This study innovatively unveiled that exposure to EE2 and elevated temperature induces adaptive hepatocytic changes, which adversely affect guppy growth and liver size, while reinforcing the species as a valuable model for assessing multiple environmental stressors.

## 2. Materials and Methods

### 2.1. Animals and Husbandry

Adult ornamental guppy males were obtained from a licensed local trader. Once they arrived at the laboratory, they were randomly allocated in triplicate to 20 tanks, each containing 5 L of dechlorinated and well-aerated freshwater. From a baseline temperature of 27.5 °C, the animals were acclimated for 20 days at 26 or 29 °C. The temperature was kept steady using high-quality individual submersible heaters (Aquael Gold, Warsaw, Poland). The temperature was raised or lowered by 0.5 °C a day until the intended values were reached. The daily temperature variation was maintained within a short range of ±0.5 °C. The photoperiod was kept at 12 h dark and 12 h light during acclimation and the experiment. 

Each aquarium had a multi-function mini submersible filter with an internal sponge. Oxygenation was continuously provided with air stones. Water quality parameters were checked every other day, and the temperature was checked twice daily. During acclimation and the experiment (in which filtering was stopped), the fish were fed twice daily with high-quality flakes for guppies (Prodac, Padua, Italy); feed uptake occurred within 30 s. At the onset of exposure (Section 2.2), the fish were weighed, and the daily feeding rate was adjusted to 2% of their body mass [32]. There were no differences between treatments (Section 2.2) as to body mass (0.31 ± 0.03 g). Throughout the study, food was consistently consumed within 30 s in all groups, suggesting no signs of feeding inhibition.

### 2.2. Exposure and Sampling

After acclimation, the animals were subjected to one of four conditions for 45 days. On day 1, each aquarium was randomly assigned to one treatment: (a) Control 26 °C; (b) Control 29 °C; (c) EE2 26 °C; (d) EE2 29 °C. Because each experimental group had five tanks, the latter is the statistically independent unit; therefore, an N of 5 was used in the Results.

In Controls, the water had ethanol (the solvent) at a nominal concentration of 0.01%, a physiologically safe level far lower than the estimated 0.25/0.50% toxicity threshold for the species [33] and the 0.20/0.50% levels known to cause ethanol-evoked liver disease in zebrafish [34,35]. In both EE2 groups, in addition to ethanol at 0.01%, the water contained EE2 at a nominal concentration of 5 ng/L. To maintain suitable water quality and constant EE2 concentrations, the water in each tank was changed every 48 h. During each change, 90% of the tank water was gradually siphoned out using a silicone tube and subsequently replaced to restore nominal concentration. This was reached by serial dilution from a stock ethanolic solution of EE2 (Sigma-Aldrich, Steinheim, Germany).

On the morning of the 46th day, each animal was killed by an overdose (2 mL/L) of 2-phenoxyethanol (Merck, Darmstadt, Germany). The body mass (BM), standard length (SL), and total length (TL) were recorded. Fulton’s condition factor (K) was computed as a proxy for guppy fitness and stoutness [36,37]: K = (100 × BM ÷ TL^3^), where BM is measured in g and TL is in cm. Before fixation for histology, a longitudinal incision was made along the fish’s ventral surface, while the caudal part was sliced off just after the end of the visceral cavity.

### 2.3. Histological Processing, Routine Staining, and Glycogen Detection in Hepatocytes

Each fish was immersed in toto in Bouin’s fixative at room temperature (ca. 20 °C). The fixation lasted 48 h, after which the fish was washed twice and kept in 70% ethanol. Before further processing, the head was severed with care not to damage the visceral cavity. An automatic tissue processor (Leica TP1020, Wetzlar, Germany) was used to handle the remaining body. The fixed pieces were immersed in an ascending series of alcohols (Proclínica, Portugal) and then cleared in xylene (VWR, Rosny-sous-Bois, France). The protocol consisted of 2 h of serial baths in ethanol (70%; 90%; 2 × 95%), followed by 2 h and 3 h in absolute ethanol and then 2 h in ethanol-xylene, 2 h and 3 h in xylene, and 2 × 3 h in paraffin. This in-house protocol offered optimal quality. The samples were embedded in paraffin (Shandon, Thermo Scientific, Waltham, MA, USA) using an embedding station (Leica EG1140H, Wetzlar, Germany); each fish was positioned to obtain cross-sections, starting from the most anterior part of the animal.

The paraffin sections were obtained with disposable blades (Feather, Japan) in a fully motorized rotary microtome (Leica RM2155, Wetzlar, Germany). Each block was serially sectioned over a body length range that comfortably covered the liver at a nominal thickness of 25 μm. Using thick sections does not compromise general histological study but is required to implement the optical disector stereological probe to obtain unbiased estimates. Four equally oriented sections were mounted on silane-coated glass slides (VWR Microscope Slides, Avantor, Radnor, PA, USA). Sections were dewaxed, rehydrated, and stained. The sections containing the liver were considered for the detailed study; testes were also examined, albeit complementarily. The majority were stained with Mayer’s haematoxylin (Merck, Germany) and eosin Y 1% aqueous (Merck, Germany), but at least one slide per fish was stained for periodic acid-Schiff (PAS) to expose polysaccharides. The protocol consisted of placing in 0.5% periodic acid for 20 min, washing in tap water for 5 min, rinsing in distilled water, placing rehydrating sections in Schiff reagent (Thermo Scientific, USA) for 30 min, washing in tap water for 5 min, counterstaining in Mayer’s haematoxylin for 1 min, washing in tap water for 5 min, dehydrating, and coverslipping. Coverslipping was performed with a synthetic medium (Q Path Coverquick 2000 medium, VWR, Radnor, PA, USA).

### 2.4. Qualitative and Semiquantitative Analysis of the Hepatocytes

The hepatocytes were observed using a light microscope (Olympus BX50, Tokyo, Japan) equipped with high-quality objectives, including a higher numerical aperture (1.35) × 100 immersion lens, and photographs were taken with a digital camera (EP50, Olympus, Tokyo, Japan). To semiquantitatively evaluate the extent of cytoplasmic vacuolation in hepatocytes per fish, the serial section presenting the largest liver area was chosen. A semiquantitative scale, graded from 0 to 3, was determined, adapting a scheme previously used in fish liver [38]. Grade 0 corresponds to hepatocytes without vacuolar aspect; grade 1 to those containing few vacuoles that occupy less than half of the cytoplasm; grade 2 when presented with many vacuoles that occupy about half of the cytoplasm; grade 3 relates to hepatocytes containing many vacuoles and that occupy almost all the cytoplasm. Author Margarida Vilaça (earlier trained junior researcher) and Eduardo Rocha (senior researcher) attributed the rating. The junior made the initial evaluation, which was later reviewed by the senior (blindly). In the event of disagreement, the slides of the animal were jointly re-evaluated until a consensus was reached. The interobserver agreement rate was 95%.

### 2.5. Lipid Droplets Visualization in Hepatocytes

A lipid-specific post-fixation and visualization protocol [39,40] was applied to supplementary control and EE2-exposed fish. Bouin fixed body fragments containing the liver were washed for 10 min in tap water under agitation. Then, they were post-fixed for 8 h under agitation in a mixture of 2.5% potassium dichromate (BDH Chemicals Ltd., Poole, UK) and 1% osmium tetroxide (Agar Scientific, Stansted Mountfitchet, UK) in 0.1 M sodium cacodylate buffer (Merck, Germany). The fragments were then washed in water for 10 × 10 min under agitation and then processed in an automated tissue processor (TP1020, Leica, Germany). The processing protocol consisted of serial 1 h baths in ethanol (70%, 90%, and 2 × 95%), followed by 1 h in absolute ethanol, then 1 h in ethanol-xylene, 1 h in xylene, and 2 × 1 h in paraffin. The fragments were then embedded in paraffin (Shandon, Thermo Scientific, USA) using an embedding station (EG1140C, Leica, Germany) and sectioned in a fully motorized rotary microtome (RM2155, Leica, Germany) with a thickness of 3 µm. The sections were mounted on silane-coated slides (VWR Microscope Slides, Avantor, USA). After 1 h at 60 °C, they were dewaxed and coverslipped with mounting medium (Q Path Coverquick 2000 medium, VWR, Avantor, USA) without staining.

### 2.6. Stereological Analysis and Derived Quantitative Parameters

Design-based stereological techniques—that is, both unbiased and accurate, as they do not depend on assumptions about the particles’ size, shape, or spatial orientation [41,42,43,44]—were used to estimate absolute quantities: the number of hepatocytes (N_Cell_) and the volumes of the liver (V_Liver_) and of the cell (V_Cell_) and nucleus (V_Nuc_) of hepatocytes. Those techniques were implemented with the help of a stereological workstation controlled by the software CAST-Grid 1.5 (Olympus, Søborg, Denmark A/S), which enabled field sampling, counting, and measurement. These procedures were performed on live images captured by a CCD camera (Sony, Tokyo, Japan)—coupled to a light microscope (Olympus BX50, Tokyo, Japan) with a high numerical aperture ×100 oil immersion lens (NA = 1.35)—and displayed on a 17’’ monitor (Samsung, Seoul, Republic of Korea).

To apply the fractionator principle [41], all the sections of each fish were subjected to systematic uniform random sampling (SURS). The spacing between the sampled sections, dependent on the size of the animals, was performed to obtain, on average, 11 sections per liver per fish for analysis—a number in line with the theoretical framework. For example, in a small fish, one was selected in every 8 sections, while in a larger one, one was chosen in every 24 sections. Differential sampling between fish is accounted for in the optical fractionator. SURS was also performed throughout the sections using x–y stepwise displacements (accuracy: 1 μm) of 325, 375, or 425 μm with the help of a motorized stage (Prior, Rockland, MA, USA). A digital length gauge linear encoder (Heidenhain MT-12, Traunreut, Germany) controlled and informed the software of the z-axis movements (accuracy: 0.5 μm).

In every sampled field, a 3D probe, the optical disector [45], was implemented. This probe included a counting frame with inclusion and exclusion lines to avoid edge effects [46], which had an area of 202.65 μm^2^ and a height of 12 μm. Upper and lower guard areas with guard zones of 4 μm were used.

The optical fractionator principle—combining the fractionator and optical disector principles—allowed us to estimate the N_Cell_ through the following formula, as previously recommended for the liver by Marcos et al. (2006) [44]:

N_Cell_ = (1 ÷ *bsf*) × (1 ÷ *ssf*) × (1 ÷ *asf*) × (1 ÷ *hsf*) × ∑ Q^−^, (1)

where *bsf* stands for block sampling fraction (*bsf* = 1, because the liver was not sampled before paraffin embedding); *ssf* means section sampling fraction (which varied according to each animal size); *asf*, area sampling fraction, is defined as the area of the frame divided by the area associated with each x, y movement; *hsf* stands for height sampling fraction, i.e., the height of the disector divided by the mean section thickness for each; finally, ∑ Q^−^ corresponds to the cells counted on the whole liver.

The cells were only counted when the nucleus was within the counting frame or touching the inclusion lines but not the forbidden lines and the nucleolus was in focus along the disector height; if the nucleolus was imperceptible, it was considered a virtual point at the center of the focused nucleus; this assumption was previously validated and successfully applied [47,48].

Compliance with these requirements was also essential to estimate the V_Cell_ and V_Nuc_ using the nucleator method [42,43]. Four orthogonally oriented lines were created from the nucleolus (the sampling point), and the interception of these lines with the cellular membrane and the nuclear envelope was pointed out. The software measured the lengths from the nucleolus to those interceptions, deriving the V_Cell_ and V_Nuc_ through the formula:

(2) $$V_{\mathrm{Cell}}\;\mathrm{or}\;V_{\mathrm{Nuc}}\;=(4\pi \;÷\;3)\;\times \;\bar{l^{3}},$$

where $\bar{l}$ represents the mean lengths measured on each estimate (here, 4).

Employing the Cavalieri principle [49], the V_Liver_ was estimated by the formula:

V_Liver_ = Distance between the analyzed sections × ∑ Liver area per section, (3)

The distance between the sections is given by the expression:

Sections’ mean thickness × (1 ÷ *ssf*). (4)

The liver area was estimated by applying the points’ differential counting technique, according to the Delesse principle [50]. The image of each sampled section—captured by a digital camera (EP50, Olympus, Japan) coupled to a light microscope (BX50, Olympus, Japan)—was overlaid with a 10 × 10 square lattice grid comprising 100 points (which had an associated area of 53,520 µm^2^); the points that appeared within the liver were counted, and its sum was multiplied by the point associated area, giving the total area of the liver per fish.

From the primary absolute parameters—V_Liver_, V_Cell_, V_Nuc_, and N_Cell_—relative quantities were derived: the number of cells per liver volume [N_V_ (Cell, Liver)], the nucleus/cell volume ratio [V_V_ (Nuc, Cell)], and the nucleus/cytoplasm volume ratio [V_V_ (Nuc, Cyto)], by the following formulas:

N_V_ (Cell, Liver) = N_Cell_ ÷ V_Liver_ (5)

V_V_ (Nuc, Cell) = V_Nuc_ ÷ V_Cell_ (6)

V_V_ (Nuc, Cyto) = V_Nuc_ ÷ (V_Cell_ − V_Nuc_). (7)

As one additional and final relative stereological parameter, the number of cells per unit volume of parenchyma [N_V_ (Cell, Par)] was estimated directly as follows:

(8) $$N_{V}\;(\mathrm{Cell},\;\mathrm{Par})=\frac{number\;of\;cells\;counted\;per\;disector}{volume\;of\;the\;disector\;({mm}^{3})}=\frac{number\;of\;cells\;counted\;per\;disector}{frame\;area\times disector\;heigth\;\left({mm}^{3}\right)}$$

Finally, taking into account the V_Liver_ and the BM, the (HSI) was estimated for every fish with the following formula:

HSI = 100 × V_Liver_ ÷ BM (9)

The HSI was estimated as a % of volume per unit mass; note that the liver density is ≈1.0 from fish [51] to humans [52], and thus, the estimated HSI is roughly equivalent to that derived from the % ratio of liver mass to body mass.

### 2.7. EE2 Quantification in Water

To confirm EE2 concentrations and absence in the solvent control, chromatographic quantification was performed on weekly water samples over 6 weeks. Each sample was 1 L (with 3 replicates per group) and placed in clean amber flasks. The collection was made before and after water renewal, and the average values per group were computed across all samples in each condition. The samples were filtered using 0.45 mm glass fiber filters (Millipore, Cork, Ireland) to remove suspended particles and solids. All the filtrates were acidified to pH 2 with H_2_SO_4_ (Sigma-Aldrich, Steinheim, Germany) and stored at 4 °C before solid-phase extraction (SPE) within 48 h. The SPE was preceded by a series of steps necessary for the quantification of EE2 by GC-MS. The gas chromatograph used was a GC TRACE 1310 (Thermo Finnigan Electron Corporation, Waltham, MA, USA) coupled with an ion trap mass spectrometer (Thermo Scientific ISQ-LT GC-MS), an autosampler (Thermo Scientific Injector Triplus 100LSTM) and a TR5MS capillary column (30 m × 0.25 mm i.d., 0.25 µm film thickness). All the procedures used were previously validated and published in absolute detail [53,54].

### 2.8. Statistical Analysis

Data sets are shown as group mean, followed by standard deviation (SD) and variation coefficient (CV = SD/mean). The open-source software Jamovi (Version 2.3) was used for inferential analysis. Normality and homogeneity of variance were confirmed for each group by the Shapiro–Wilk W and the Levene tests, respectively. The significance of the difference between group means was tested by two-way ANOVA; if significant, it was followed by post hoc Tukey testing. When the interaction was non-significant, the focus fell on the independent effects. The significance of the difference between the medians of hepatocyte vacuolation degrees was analyzed using a non-parametric one-way ANOVA (the Kruskal–Wallis test) to look for significant differences; values were additionally transformed into ranks, which could be examined by a two-way ANOVA. The Chi-Square Test was used to test the % of fish survival. Statistical significance was defined as *p* < 0.05.

## 3. Results

### 3.1. EE2 Concentrations in the Water

EE2 was untraceable in the Control groups. In contrast, the mean EE2 concentration in the exposed groups was 4.85 ng/L (SD = 0.24 ng/L; CV = 4.9%). No significant differences existed between the EE2 groups at 26 °C and 29 °C.

### 3.2. Survival and Body Biometry

The survival rates were 95% (Control, 26 °C and 29 °C), 100% (EE2, 26 °C), and 87% (EE2, 29 °C), with no significant differences between groups (χ^2^ = 0.074, *p* > 0.05). The biometric results for each experimental group after the assay are given in Table 1.

**Figure 1:**
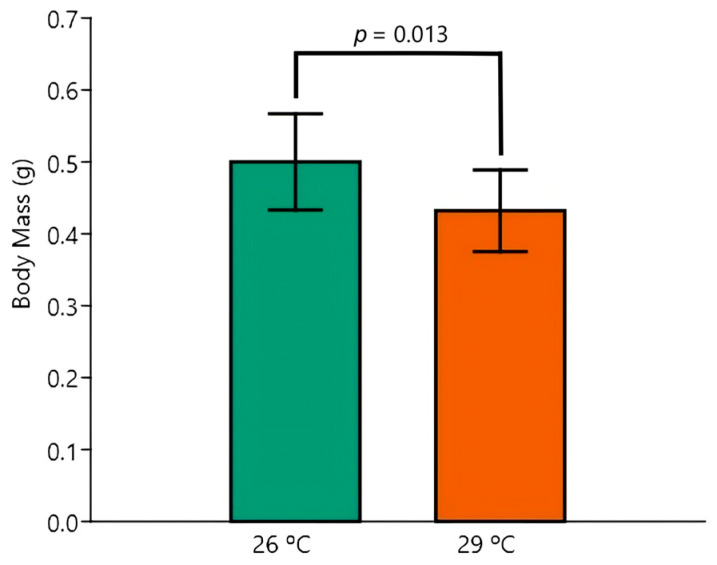
Body mass (g) of fish exposed to 26 °C or 29 °C. The two-way ANOVA revealed a significant difference between temperatures. Data are shown as mean and standard deviation (SD).

**Figure 2:**
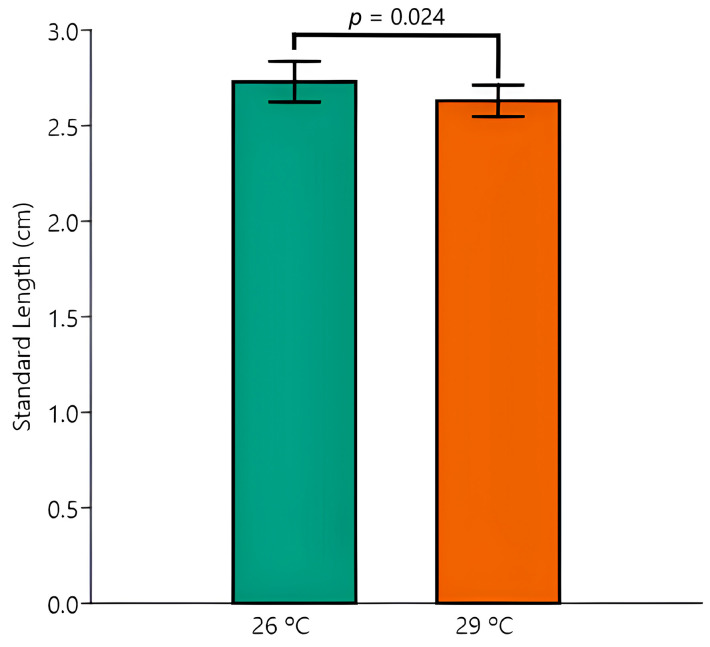
Standard length (cm) fish exposed to 26 °C or 29 °C. The two-way ANOVA revealed a significant difference between temperatures. Data are shown as mean and standard deviation (SD).

The two-way ANOVA revealed no impact of temperature, EE2 exposure, or their interaction on the parameters TL and K. In contrast, significant temperature-induced changes were observed in BM (*p* = 0.013) and SL (*p* = 0.024), with overall relative decreases in BM (Figure 1) and SL (Figure 2) in animals kept at 29 °C.

**Figure 3:**
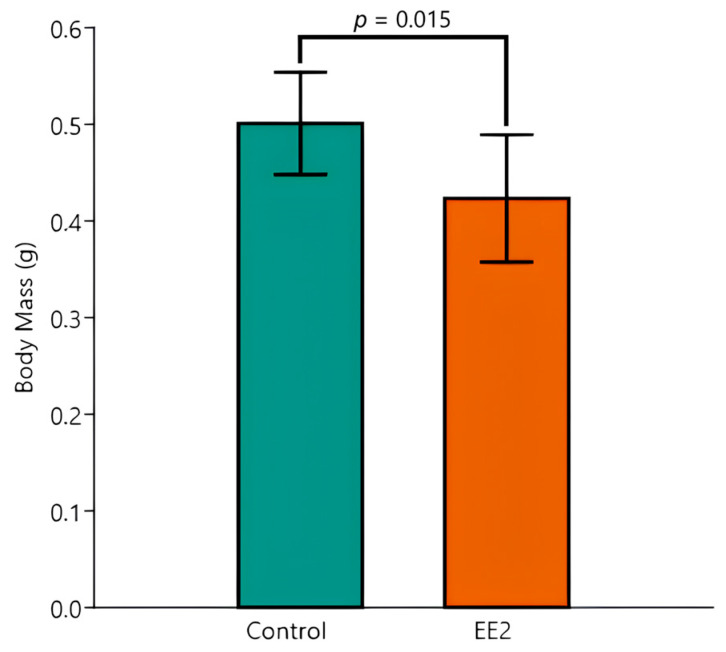
Body mass (g) of Control versus EE2 exposed fish. The two-way ANOVA revealed a significant difference in the factor group. Data are shown as mean and standard deviation (SD).

There was also an independent effect of EE2 exposure, which significantly decreased the BM when compared to the control (*p* = 0.015) (Figure 3).

Despite there being no significant interaction between the effects of temperature and EE2 exposure, the post hoc Tukey test disclosed one pairwise difference between the most extreme mean values of BM (Control 26 °C versus EE2 29 °C, *p* = 0.006) and of SL (Control 26 °C versus EE2 29 °C, *p* = 0.027).

### 3.3. Qualitative and Semiquantitative Histological Analysis

**Figure 4:**
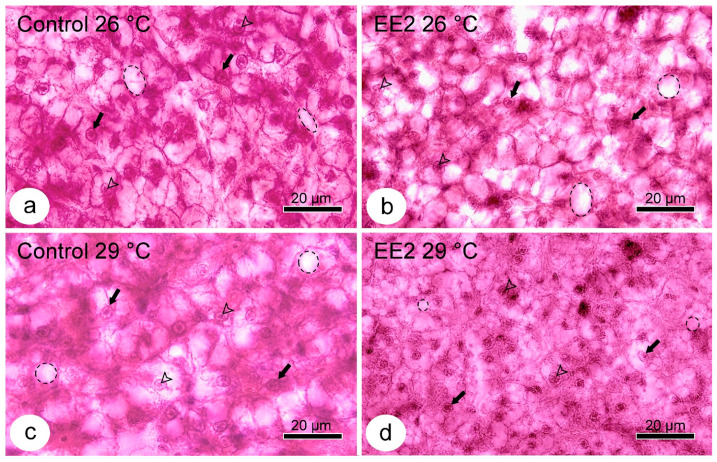
HE staining—representative images (from 25 µm thick sections) of liver parenchyma of guppy from Control 26 °C (**a**), EE2 26 °C (**b**), Control 29 °C (**c**), and EE2 29 °C (**d**). Note the vacuolated aspect of the hepatocytes from Controls and the EE2 26 °C group and how the pattern in the EE2 29 °C looks different from the others, with hepatocytes having fewer and smaller vacuolar spaces. Nucleus: arrow; Nucleolus: arrowhead; Cytoplasmic vacuolar space: circle and ellipse.

The analysis of liver microanatomy revealed no histopathological lesions in the stroma or parenchyma across all four experimental conditions. Regarding the hepatocytes, despite being within the species’ normal range (Figure 4), a notable characteristic was the vacuolated cytoplasm.

The extent of vacuolation was semi-quantified, and the results are summarized in Table 2. The Control and EE2 exposed groups, held at 26 °C and the Control at 29 °C, exhibit a similar vacuolation pattern, with equal median and mode values. The group EE2 at 29 °C stands out due to its lower median and mode values. The Kruskal–Wallis test revealed a significant difference between groups (*p* = 0.013). Furthermore, the two-way ANOVA of the ranked scores revealed a significant difference between the Control and EE2-treated groups (*p* = 0.009), independent of temperature. The Tukey test further indicated a significant difference between the Control at 29 °C and EE2 at 29 °C (*p* = 0.036).

### 3.4. PAS Staining

**Figure 5:**
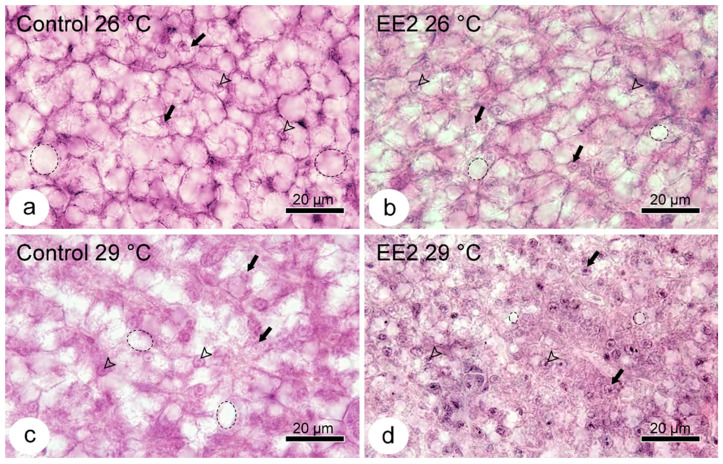
PAS staining—images (from 25 µm thick sections) illustrating the liver parenchyma of guppy from Control 26 °C (**a**), EE2 26 °C (**b**), Control 29 °C (**c**), and EE2 29 °C (**d**). Note the pale and colorless appearance of the vacuolated cytoplasm, indicating that the whitish spaces had no glycogen and should have resulted from the extraction of lipid droplets. Nucleus: arrow; Nucleolus: arrowhead; Cytoplasmic vacuole: circle and ellipse.

The PAS staining was performed to determine the glycogen (which is PAS-positive) content and distribution in hepatocytes, as well as whether the vacuolated cytoplasm was due to glycogen or, by exclusion, to lipid deposits. Whenever present, the latter ones are extracted during complete dehydration, and the areas they occupy in vivo look unstained with HE or PAS. In this study, the hepatocytes were virtually devoid of glycogen deposits in all the assayed conditions, and the vacuolar spaces did not stain with PAS (Figure 5).

### 3.5. Lipid Droplets Staining

**Figure 6:**
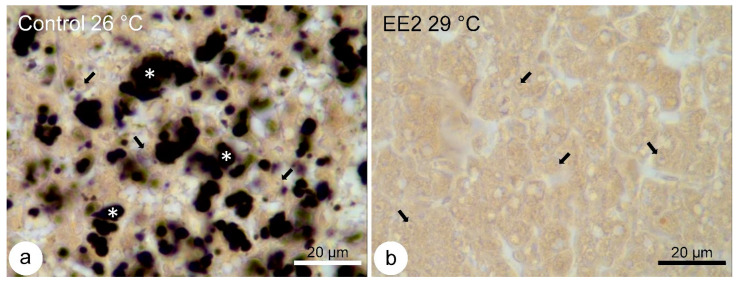
Osmium tetroxide post-fixation—images (from 3 µm thick sections) illustrating the liver parenchyma of guppy from Control 26 °C (**a**) and EE2 29 °C (**b**); the image in (**a**) is representative of the parenchyma seen in Control 29 °C and EE2 26 °C. Observe in (**a**) the darkly stained intercellular groups of lipid droplets (white asterisks) in contrast with their absence in (**b**). Nucleus: arrow.

The osmium tetroxide post-fixation protocol, allowing univocal visualization of cytoplasmic lipid droplets, was used to further investigate if the vacuoles seen in HE- or PAS-stained sections were due to lipid deposits. The observations confirmed that vacuolation was primarily caused by the hepatocyte lipid droplet load, and that the group with less vacuolation (EE2, 29 °C) was evidently the one with fewer or no droplets (Figure 6).

### 3.6. HSI and Stereological Parameters

**Figure 7:**
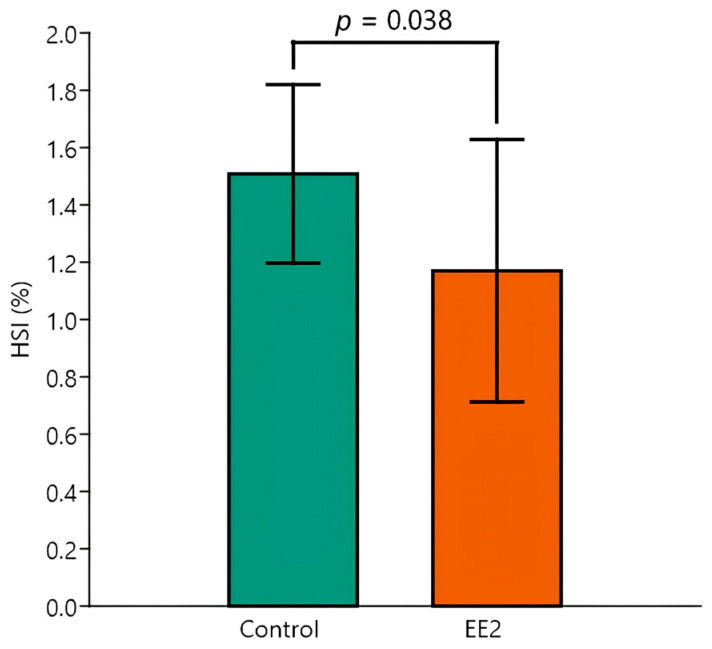
Hepato-somatic index (HSI) of Control versus EE2 exposed fish. The two-way ANOVA revealed a significant difference in the factor group, irrespective of temperature. Data are shown as mean and standard deviation (SD).

Regarding the HSI, the data are presented in detail in Table 3. Like the V_Liver_, the two-way ANOVA showed a significant effect of the EE2 exposure factor, with control animals having a higher mean HSI than those exposed to EE2 (*p* = 0.038) (Figure 7). Tukey’s post hoc testing flagged a significant pairwise difference between the Control 29 °C versus EE2 groups at 29 °C (*p* = 0.04). There was no significant effect of temperature and interaction with EE2 exposure. Despite HSI being connected with BM, the inter-animal variability of the latter was overall greater than that of BM, with CVs ranging from ≈ 11% to 35%.

The absolute parameters V_Liver_, V_Cell_, V_Nuc_, and N_Cell_ are presented in Table 4. The relative ones, N_V_ (Cell, Liver), N_V_ (Cell, Par), V_V_ (Nuc, Cell) and V_V_ (Nuc, Cyto), are listed in Table 5.

**Figure 8:**
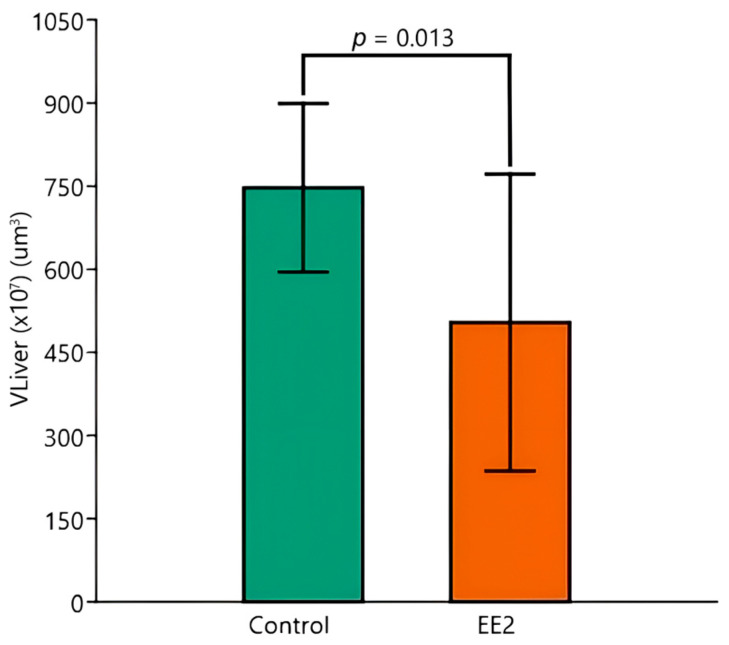
Liver volume (V_Liver_) (µm^3^) of Control versus EE2 exposed fish. The two-way ANOVA revealed a significant difference in the factor group, irrespective of temperature. Data are shown as mean and standard deviation (SD).

Regarding the V_Liver_, the two-way ANOVA evidenced a significant overall effect of EE2 exposure, with control animals having a higher mean V_Liver_ than EE2-exposed ones (*p* = 0.013) (Figure 8). The post hoc Tukey test reinforced the result, illustrating specific pairwise differences: Control 26 °C versus EE2 29 °C (*p* = 0.033) and Control 29 °C versus EE2 29 °C (*p* = 0.029). Despite the higher temperature seeming to reinforce the effect of EE2 on organ size, the ANOVA did not reveal significant effects of temperature or interaction between temperature and group exposure conditions. The inter-animal variability was generally high, except for the Control group at 29 °C, which had a CV below 10%.

**Figure 9:**
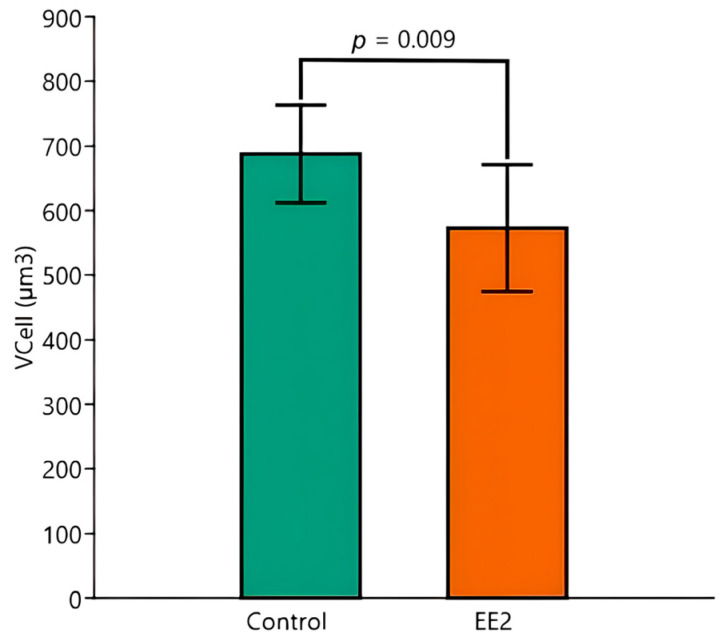
Hepatocyte volume (V_Cell_) (µm^3^) of Control versus EE2 exposed fish. The two-way ANOVA revealed a significant independent effect of the factor exposure, regardless of temperature. Data are shown as mean and standard deviation (SD).

Concerning V_Cell_, the two-way ANOVA indicated that exposure to EE2 significantly decreased it (*p* = 0.009) (Figure 9). A post hoc Tukey test to identify synergies between the temperature and exposure to EE2 showed significant differences between Control 29 °C versus EE2 29 °C (*p* = 0.039) and Control 26 °C versus EE2 29 °C (*p* = 0.019). As for the V_Liver_, temperature seems to strengthen the effect of EE2. However, the ANOVA did not disclose a significant temperature impact or an interaction between the two tested factors. The V_Cell_ had a lower variability than V_Liver_, with CV values ranging from ≈ 7% to 16%.

**Figure 10:**
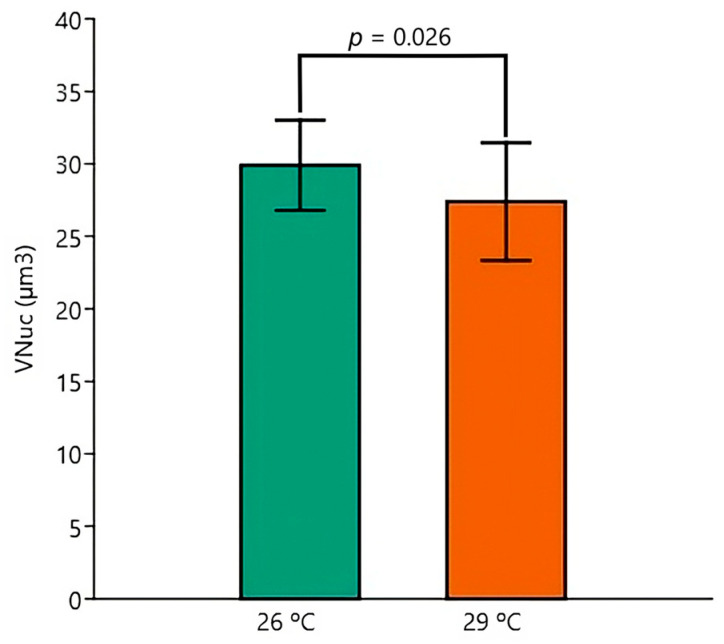
Nuclear volume of hepatocytes (V_Nuc_) (µm^3^) of fish kept at 26 °C or 29 °C. The two-way ANOVA revealed a significant independent difference between temperatures. Data are shown as mean and standard deviation (SD).

**Figure 11:**
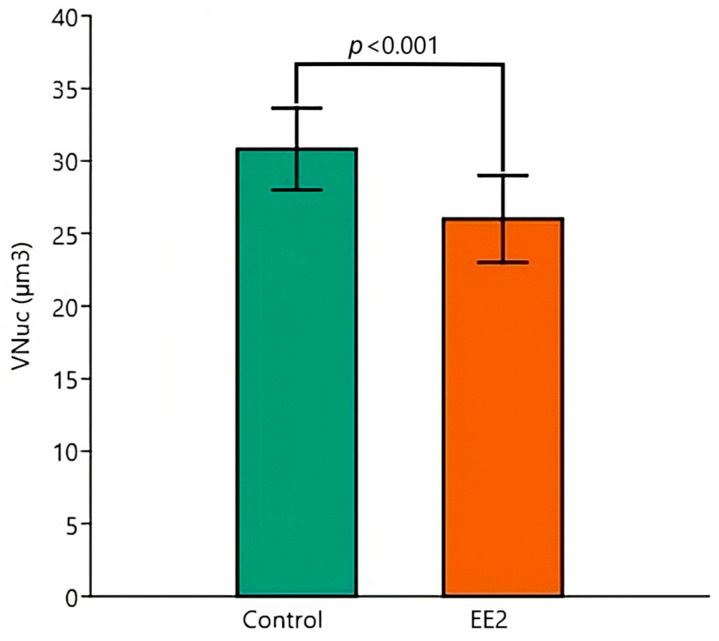
Nuclear volume of hepatocytes (V_Nuc_) (µm^3^) of Control and EE2 exposed fish. The two-way ANOVA revealed a significant independent effect of EE2 exposure. Data are shown as mean and standard deviation (SD).

Respecting the V_Nuc_, two-way ANOVA revealed independent significant effects of temperature (*p* = 0.026) (Figure 10) and exposure condition (*p* < 0.001) (Figure 11), with the 29 °C and EE2 leading to reductions in the mean values. Tukey’s post hoc tests identified significant pairwise differences between Control 26 °C versus EE2 29 °C (*p* < 0.001), Control 29 °C versus EE2 29 °C (*p* = 0.001) and EE2 26 °C versus EE2 29 °C (*p* = 0.034).

As to the N_Cell_, the ANOVA did not flag significant differences. The N_Cell_ presented high variability in all the experimental groups, with CV values consistently above 20%.

Regarding the relative stereological parameters (Table 5), the two-way ANOVA did not reveal any significant differences, indicating that the mean values were not influenced by the tested temperatures or exposure to solvent versus EE2. The V_V_ (Nuc, Cell) and V_V_ (Nuc, Cyto) had consistent mean values across groups. Overall, the CVs per group were lower for the relative volumes than for the numerical densities; the Control at 29 °C exhibited the lowest variability, while the other groups showed overall equal variability.

### 3.7. Testis Histology

**Figure 12:**
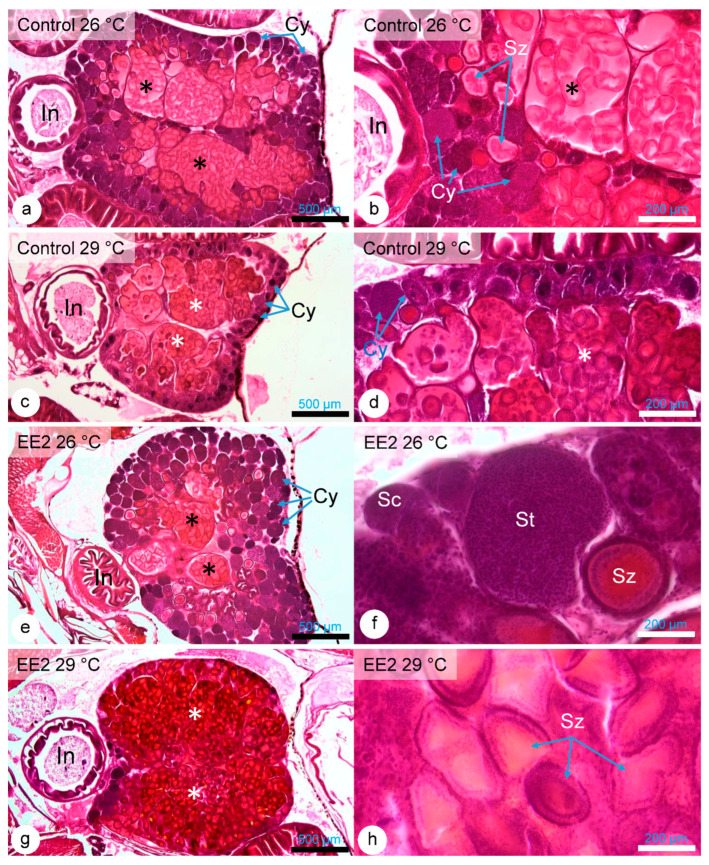
HE staining—images illustrating the diversity of histological aspects and maturation degrees of guppy testis from Control 26 °C (**a**,**b**), Control 29 °C (**c**,**d**), EE2 26 °C (**e**,**f**), and EE2 29 °C (**g**,**h**). Asterisks (black/white): spermatozeugmata in efferent ducts; Cy; Cysts (spermatocysts); In: Intestine; Sc: Spermatocytes within spermatocysts; St: Spermatids within spermatocysts; Sz: spermatozeugmata.

Complementarily, an overview of the testis maturation status was made to histologically confirm that the animals were adults and to identify any potential qualitative differences between the experimental groups. It was confirmed that despite some expected inter-individual variability, all fishes exhibited active spermatogenesis and displayed all the spermatogenesis stages, including the formation of the typical spermatozeugmata (circular arrays of spermatozoa encircled in a carbohydrate-rich sheath). A comparative detailed overview of the animals of the different groups did not reveal differences that could be attributed to a specific exposure condition. The variability of testicular aspects is presented in Figure 12, including the fact that even animals of the EE2 29 °C could display a fully mature testis.

## 4. Discussion

This study utilized morphometrics, histology, and design-based stereology to evaluate the effects of EE2 and supra-optimal temperature on male guppy biometry, liver volume, hepatocyte size and cellularity, and hepatocyte glycogen and lipid reserves. Combined, those factors reduced body mass growth and liver volume, mainly by reducing cell size and lipid reserves, not cell number. Mortality and spermatogenesis were unaffected.

Regarding the temperature, our choice was based on worst-case climate predictions, which anticipate an increase of up to 4 °C by 2100 [55]. We also considered that guppy’s natural habitats present temperatures between 24 and 30 °C [56], with 26 °C being optimal for the species’ reproduction. Metabolic costs should increase at 29 °C, which falls in the upper limit for this species. Fish exposed to increased temperatures may become nutrient-depleted due to greater energy demands from increased metabolism [57]. In the present study, fish from the control and exposed groups were fed similarly. Therefore, the observed lower BM and SL at 29 °C was probably due to the higher basal metabolic rate associated with coping with the increased temperature. Similarly to our results, reduced body growth was also observed in the red moki (*Cheilodactylus spectabilis*) when exposed to temperatures above its optimal 16 °C [5] and in the Antarctic eelpout (*Pachycara brachycephalum*) exposed to 5 and 10 °C [58], with growth decreasing at the higher temperatures.

Regarding the EE2, our option (≈5 ng/L) aligns well with environmentally relevant concentrations and that may impact fish. For example, Portuguese surface waters have been reported for a long time to have average concentrations of EE2 of either ≈10 ng/L [21] or ≈7 ng/L [15]. However, worldwide, the concentrations of EE2 in water vary considerably, ranging, when detected, from 0.1 to over 60 and, more rarely, even over 100 ng/L [15,21]. Such concentrations pose risks of disruption for aquatic biota, namely fish as vertebrates. Overall, the 45-day exposure to EE2 in this study significantly comparatively reduced the BM, hepatocyte vacuolation, HSI, V_Liver_, V_Cell_, and V_Nuc_ of the adult male guppies.

Corroborating our finding that EE2 can reduce BM gain, a 30-day dietary exposure to E2 decreased muscle growth and BM in sterile female rainbow trout [59]. Guppy growth was also inhibited by a 91-day exposure to 0.1–10 µg/L of the xenoestrogen diethylhexyl phthalate (DEHP) [37]. In another study, zebrafish juveniles exposed to EE2 at 1 and 10 ng/L for 33 days showed no difference in BM, but 100 ng/L reduced the BM [60]. On the contrary, yellow catfish (*Pelteobagrus fulvidraco*) exposed for 56 days to a higher level of DEHP (1.0 mg/L) and a mixture of DEHP and EE2 (1.0 mg/L and 1.0 µg/L, respectively) gained weight, relative to the control [61]. Overall, although EE2 and other xenoestrogens can impact BM in guppy and other fish, it is not advisable to generalize across species. Studies on distinct fish groups are warranted for more accurate predictions.

The EE2-exposed group at 29 °C presented less hepatocyte vacuolation. In contrast, the two groups at 26 °C and the Control at 29 °C had a similar pattern. Therefore, that decrease only occurred due to the combined action of both stressors. Additionally, the vacuoles did not stain with PAS, excluding the presence of glycogen deposits and supporting that those vacuoles (appearing as unstained areas in HE) corresponded to lipid droplet storage in vivo. A post-fixation technique with osmium tetroxide confirmed that the EE2 29 °C group had rare lipid droplets in hepatocytes. Note that the highly vacuolated aspect of hepatocytes in the Control fish was somewhat expected since fish livers are commonly more vacuolated than those of mammals [62]. This is particularly true for captive fish, which tend to eat more and expend less energy than their wild counterparts [62,63]. Consistent with our observations, vacuolated, lipid-laden hepatocytes have been previously reported in normal, healthy guppies [64]. Despite decreasing glycogen and/or lipids in the fish liver being a common sign of toxicity, the opposite can happen [62]. Herein, decreased vacuolation and lipid droplets likely reflect increased liver nutrient mobilization to meet the metabolic demands imposed on the guppies by both stressors.

Whereas estrogens are more closely linked to lipid metabolism [65], temperature affects metabolism more broadly [66]. These influences on metabolism can impact morphology, either increasing or decreasing the vacuolation of hepatocytes. For example, contrary to our results, after a 48 h exposure to hyperthermia (32 °C, compared to 25 °C), the Wuchang bream presented hepatocytes richer in larger fat droplets [12]. This increase in lipid content (and vacuolation) was seen as an adaptation that enhances cellular resistance to higher temperatures. Indirectly aligning with our findings, the lipidic contents of catfish (*Ictalurus punctatus*) liver decreased in the summer (when the temperatures were higher); however, contrary to our study, the HSI decreased [67]. Our data further support that fish species differ in hepatocytic vacuolation responses to rising temperatures. Given the limited literature on temperature effects on liver vacuolation in fish and its histopathological relevance of this condition, more studies are needed to determine how much this condition is adaptive or pathological. Additionally, research is required to understand why some species increase, while others show decrease, vacuolation as temperature rises.

Changes in the HSI have been used as a proxy of toxicant exposure in biomonitoring [68,69], with this ratio often rising in fish after exposure to estrogens [26,70]. Here, the HSI decreased with exposure to EE2. In contrast, the HSI was stable in male platy fish given 25 μg/g of EE2 [25] and in Nile tilapia (*Oreochromis niloticus*) exposed to 10–50 ng/L of E2, despite the liver lipid accumulation [71]. The latter result contradicts our findings, as EE2 decreased the hepatic vacuolation and lipid droplet load in guppies. Also, in contrast to our results, yellow catfish exposed to the xenoestrogen DEHP (1.0 mg/L) and to a mixture of DEHP and EE2 (1.0 mg/L and 1.0 µg/L, respectively) exhibited a higher HSI [61]. In the estrogen-sensitive species rainbow trout, juveniles or males exposed to either E2 or EE2 with varying doses and methods typically increase the HSI through liver hypertrophy [72,73]. This process has been linked to estrogen-stimulated vitellogenin production, with liver enlargement reflecting greater synthesis [74]. Increased HSI and liver mass were also linked to greater expression of biotransformation enzymes [75].

Here, HSI was unaffected by temperature, denoting that despite a tendency for lower BM at 29 °C, animals maintained a balance between liver and body masses, which is vital for functional optimization. However, there are reports of HSI decreasing with increased temperatures. For example, the HSI of Nile tilapia was lower at 27 °C, compared to 17 °C [76], and that of the Atlantic salmon decreased at 21 °C, compared to 15 °C [13].

Similarly to BM and HSI, the V_Liver_, V_Cell_ and V_Nuc_ decreased with exposure to EE2, while the N_Cell_ did not differ (also with temperature). The drop in V_Liver_ resulted from reduced V_Cell_ (together with V_Nuc_) rather than fewer hepatocytes, which would imply heat- or chemical-induced cell death [77,78]. No histopathological lesions were observed in the guppies exposed to EE2 at either temperature, consistent with the stable hepatocyte population across groups. Interestingly, smaller V_Nuc_ is associated with lower miotic rate [79], and significant hepatocyte proliferation occurs only in the context of liver injury [80]. After exposure to EE2 at 29 °C, the reduction in the V_Cell_ correlates with decreased cytoplasmic vacuolation and fewer lipid droplets. Lipid droplet depletion has been shown to decrease hepatocyte volume [81], a process also triggered by other catabolic changes [82].

The V_V_ (Nuc, Cell) and V_V_ (Nuc, Cyto) had consistent mean values across groups. This finding supports the idea that the guppy liver underwent an adaptive process where the hepatocytes adjusted their volume and nucleus-to-cytoplasmic ratio in response to the stressors without entering the pathological spectrum. It has been verified that nucleus-to-cytoplasmic volume ratios that do not fall within optimal limits indicate cell stress, toxicity, and pathological alterations [83,84]. Moreover, although the guppy exhibited credible cellular adaptations, these did not prevent organism-level impacts. EE2 exposure reduced both V_Liver_ and BM, disrupting their balance and lowering the HSI—meaning less liver tissue per unit of body mass. In humans, reduced liver mass is well known to correspond to diminished function, at least temporarily [85]. To our knowledge, this relationship has not been studied in fish; however, a similar scenario is reasonable to assume.

Hepatic adaptation may have supported the maintenance of other organ functions, specifically the testis, while enabling some growth and good survival (>85%), above the 80% reported in male guppies exposed for 28 days to 850 ng/L of the natural estrogen E2 [23]. In our study, histology confirmed all animals were adults and revealed that, despite some variability, all exhibited active spermatogenesis, including mature sperm cells in spermatozeugmata. No differences were found between groups. It could initially be presumed that, if not the highest temperature, EE2 exposure might have a histological impact on spermatogenesis. However, this was not the case. Our findings are consistent with previous research. In one study, adult guppy males exposed to E2 (30 and 100 ng/L) for 60 days [86] maintained all stages of spermatogenesis, with hypertrophy of Sertoli cells and efferent duct cells only at the highest concentration. In another study, adult males were exposed to very high levels of E2 (850 ng/L) for 28 days and tended to show more spermatozeugmata at the expense of losing earlier spermatogenic stages [23]. At last, newborn males exposed to EE2 (10, 50, and 200 ng/L) for 3.5 months until adulthood [87] showed a trend toward fewer advanced spermatogenesis stages only at higher EE2 levels. Only the highest levels caused significant histological effects, leading the authors to declare that “environmental estrogens do not seem to pose a reproductive threat to guppies. Although our results seem to support this conclusion, we advise caution in generalizing, since the same studies show that some xenoestrogens, other than EE2 and E2, affect the testis in the same studies, albeit typically at high, supra-environmental concentrations [86,87].

## 5. Conclusions

This study examined the effects of EE2 and supra-optimal temperature on body biometry, liver volume, and hepatocyte histology and stereology in adult male guppies. Testis maturation was also assessed to complement hepatic findings. After 45 days, higher temperature (29 °C) reduced body mass (BM) and standard length (SL) gains, likely due to increased metabolic demand, as seen in other fish under thermal stress. Unlike other species, temperature alone did not affect HSI. In contrast, EE2 (~5 ng/L) disrupted this balance, lowering HSI and BM gain and reducing hepatocyte vacuolation, V_Liver_, V_Cell_, and V_Nuc_, while N_Cell_ remained unchanged—suggesting an adaptive response without histopathological damage. Vacuolation dropped only when EE2 and high temperature combined, likely reflecting greater lipid mobilization to meet higher metabolic demands. Despite reductions in cellular and nuclear volumes, V_V_ (Nuc, Cell) and V_V_ (Nuc, Cyto) ratios stayed stable, reinforcing a non-pathological, adaptive hepatic response. Stressed guppies maintained hepatocyte number and nuclear–cytoplasmic balance, showing the liver’s capacity to adjust structurally without degeneration. All males showed active spermatogenesis. Still, systemic effects on BM, SL, V_Liver_, and HSI persisted, highlighting the need for more research into interspecies variability and sub-chronic and long-term impacts of EE2 and thermal stress, alone and combined, on fish growth and liver histophysiology. These results support the guppy as a valuable model for studying temperature and xenoestrogen effects. Future work could examine additional histological markers (e.g., oxidative stress, autophagy) and include mechanistic studies to clarify the pathways behind these findings.

## Figures and Tables

**Table 1:** Body mass (BM), total length (TL), standard length (SL), and Fulton’s condition factor (K). Data are shown as mean, standard deviation (SD), and coefficient of variation (CV). The parameters BM, TL, SL and are shown in bold.

BM	Control 26 °C (n = 5)	Control 29 °C (n = 5)	EE2 26 °C (n = 5)	EE2 29 °C (n = 5)
Mean (g)	0.53	0.47	0.47	0.39
SD (g)	0.06	0.04	0.07	0.05
CV (%)	10.8	8.5	14.9	11.9
**TL**				
Mean (cm)	3.8	3.7	3.7	3.5
SD (cm)	0.2	0.2	0.1	0.3
CV (%)	6.2	5.9	2.4	7.4
**SL**				
Mean (cm)	2.8	2.7	2.7	2.6
SD (cm)	0.1	0.1	0.1	0.0
CV (%)	3.2	3.1	2.1	1.7
**K**				
Mean (100× g/cm^3^)	1.0	0.9	1.0	0.9
SD (100× g/cm^3^)	0.1	0.1	0.1	0.2
CV (%)	10.1	14.6	15.0	23.1

**Table 2:** Hepatocyte vacuolation grading in the four experimental groups. The scores are shown as median, mode, minimum, and maximum.

Vacuolation Grade	Control 26 °C (n = 5)	Control 29 °C (n = 5)	EE2 26 °C (n = 5)	EE2 29 °C (n = 5)
Median	2	2	2	1
Mode	2	2	2	1
Minimum	1	2	0	1
Maximum	3	3	2	2

**Table 3:** Hepato-somatic index (HSI, expressed as the % of Liver Volume (V_Liver_) relative to the Body Mass (BM). Data are shown as mean, standard deviation (SD), and coefficient of variation (CV).

HSI	Control 26 °C (n = 5)	Control 29 °C (n = 5)	EE2 26 °C (n = 5)	EE2 29 °C (n = 5)
Mean (%)	1.4	1.6	1.4	1.0
SD (%)	0.4	0.2	0.5	0.3
CV (%)	27.3	11.4	33.7	35.2

**Table 4:** Liver volume (V_Liver_), hepatocyte cell volume (V_Cell_), hepatocyte nucleus volume (V_Nuc_), and total number of hepatocytes per liver (N_Cell_). Data are shown as mean, standard deviation (SD), and coefficient of variation (CV). The parameters V_Liver_, V_Cell_, V_Nuc_ and N_Cell_ are shown in bold.

V_Liver_ (×10^7^)	Control 26 °C (n = 5)	Control 29 °C (n = 5)	EE2 26 °C (n = 5)	EE2 29 °C (n = 5)
Mean (µm^3^)	753.9	762.9	657.0	375.9
SD (µm^3^)	221.6	70.0	288.0	118.6
CV (%)	29.4	9.2	43.8	31.6
**V_Cell_**				
Mean (µm^3^)	704.3	677.2	632.9	528.7
SD (µm^3^)	106.5	46.1	99.5	64.0
CV (%)	15.1	6.8	15.7	12.1
**V_Nuc_**				
Mean (µm^3^)	31.8	30.9	28.2	24.0
SD (µm^3^)	2.8	2.1	2.2	1.9
CV (%)	8.7	6.8	7.6	7.9
**N_Cell_ (×10^4^)**				
Mean	33.7	43.7	36.8	26.8
SD	9.0	15.1	11.2	6.0
CV (%)	26.7	34.7	30.5	22.4

**Table 5:** Number of hepatocytes per unit of liver volume [N_V_ (Cell, Liver)], number of hepatocytes per unit of parenchyma volume [N_V_ (Cell, Par)], nucleus/cell volume ratio [V_V_ (Nuc, Cell)], and nucleus/cytoplasm volume ratio [V_V_ (Nuc, Cyto)]. Data are shown as mean, standard deviation (SD), and coefficient of variation (CV). The parameters N_V_ (Cell, Liver), N_V_ (Cell, Par), V_V_ (Nuc, Cell), and V_V_ (Nuc, Cyto) are shown in bold.

N_V_ (Cell, Liver) (×10^3^)	Control 26 °C (n = 5)	Control 29 °C (n = 5)	EE2 26 °C (n = 5)	EE2 29 °C (n = 5)
Mean (mm^−3^)	47.1	56.8	65.0	74.9
SD (mm^−3^)	15.4	17.1	38.2	19.7
CV (%)	32.8	30.1	58.9	26.3
**N_V_ (Cell, Par) (×10^5^)**				
Mean (mm^−3^)	11.0	10.8	11.9	15.1
SD (mm^−3^)	3.3	2.4	2.8	3.2
CV (%)	29.9	22.6	23.5	20.9
**V_V_ (Nuc, Cell)**				
Mean (%)	4.6	4.6	4.5	4.6
SD (%)	0.6	0.2	0.6	0.6
CV (%)	12.1	5.1	13.6	14.0
**V_V_ (Nuc, Cyto)**				
Mean (%)	4.8	4.8	4.7	4.8
SD (%)	0.6	0.3	0.7	0.7
CV (%)	12.1	5.4	14.3	14.6

## Data Availability

The datasets are available from the authors upon reasonable request.
